# Bronchogenic Cyst in the Cervical Region: A Rare Entity – A Case Report and Review of the Literature

**DOI:** 10.7759/cureus.14413

**Published:** 2021-04-11

**Authors:** Anam Mumtaz, Rahim Dhanani, Muhammad Faisal, Hina Maqbool, Amina Iqbal khan

**Affiliations:** 1 Surgical Oncology, Shaukat Khanum Memorial Cancer Hospital and Research Centre, Lahore, PAK; 2 Histopathology, Shaukat Khanum Memorial Cancer Hospital and Research Centre, Lahore, PAK

**Keywords:** bronchial cyst, cervical cyst, bronchogenic cyst, men syndrome

## Abstract

Bronchogenic cysts originate from the tracheobronchial bud, which arises from the embryonic foregut. Congenital bronchogenic cysts in the cervical region, especially in the thyroid or perithyroidal area, are extremely rare. Moreover, distinguishing them from other cervical cystic lesions such as thyroglossal duct and branchial cleft cysts and metastatic cervical lymph nodes is difficult preoperatively. In this report, we discuss a case of a 41-year-old woman who presented to us with a history of anterior neck swelling for two weeks with occasional palpitations and bilateral flank pain. On workup, she was diagnosed as a case of multiple neuroendocrine neoplasm type 2A for which she underwent adrenalectomy first followed by total thyroidectomy with central neck dissection and parathyroidectomy. On the final histopathology specimen, an incidental bronchogenic cyst was diagnosed. A bronchogenic cyst is a rare entity, especially in the head and neck region, and can be confused with a metastatic lymph node. Diagnosis is made based on the histopathological examination, which requires surgical excision. The bronchogenic cyst should be considered in the differential diagnosis for midline and lateral neck masses.

## Introduction

Bronchogenic cysts originate from the tracheobronchial bud, which arises from the embryonic foregut [1]. It has previously been reported that bronchogenic cysts account for 10% of all mediastinal masses and are more commonly found in males [2]. In more than 50% of the patients, these cysts are located in the thorax, although they can occur in an ectopic location anywhere in the developmental pathway of the foregut. Localization of such cysts in the head and neck region is very uncommon [3].

Congenital bronchogenic cysts are extremely rare in the cervical region, especially in the area around the thyroid gland, and distinguishing them from other cervical cystic lesions such as thyroglossal duct, branchial cleft cysts, and metastatic cervical lymph nodes are difficult preoperatively due to their similar radiological features [4]. Therefore, diagnosing a bronchogenic cyst in the neck region offers a great challenge.

## Case presentation

**Figure 1:**
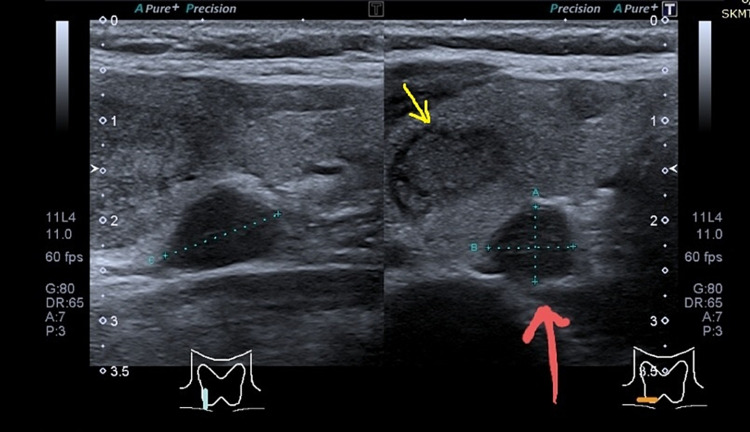
Ultrasound of the neck showing right thyroid lobe nodule (arrow in yellow) and right parathyroid gland (arrow in red)

The patient was a 41-year-old woman who presented to us with a history of anterior neck swelling for two weeks with occasional palpitations and bilateral flank pain. She had a strong family history of medullary thyroid carcinoma (MTC). A neck ultrasound was done, which showed a right thyroid lobe nodule and an enlarged right parathyroid gland (Figure 1). Fine needle aspiration cytology (FNAC) diagnosed it as MTC. On further workup, it was found that she had a left renal nodule along with renal stones. On endocrine evaluation, she was diagnosed to have multiple endocrine neoplasia (MEN) syndrome type 2A, with pheochromocytoma (left adrenal nodule), right hyper-parathyroid gland, and right MTC.

The case was discussed in the multidisciplinary tumor board (MDT), and it was decided to offer her laparoscopic adrenalectomy first, followed by total thyroidectomy with neck dissection and right parathyroidectomy.

Surgeries were performed as per the MDT's decision. The patient recovered well postoperatively without any complications. Histopathology after total thyroidectomy with neck dissection and right parathyroidectomy revealed right thyroid lobe MTC, right parathyroid adenoma, and bronchial cyst.

**Figure 2:**
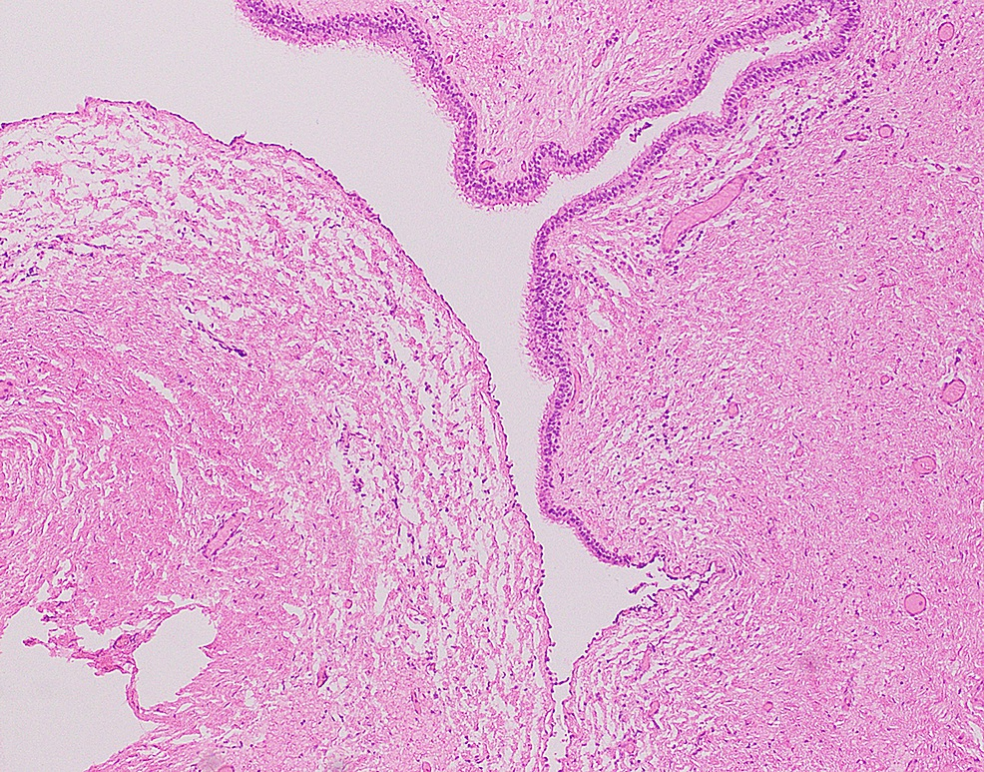
Low power view of cyst showing ciliated epithelium

**Figure 3:**
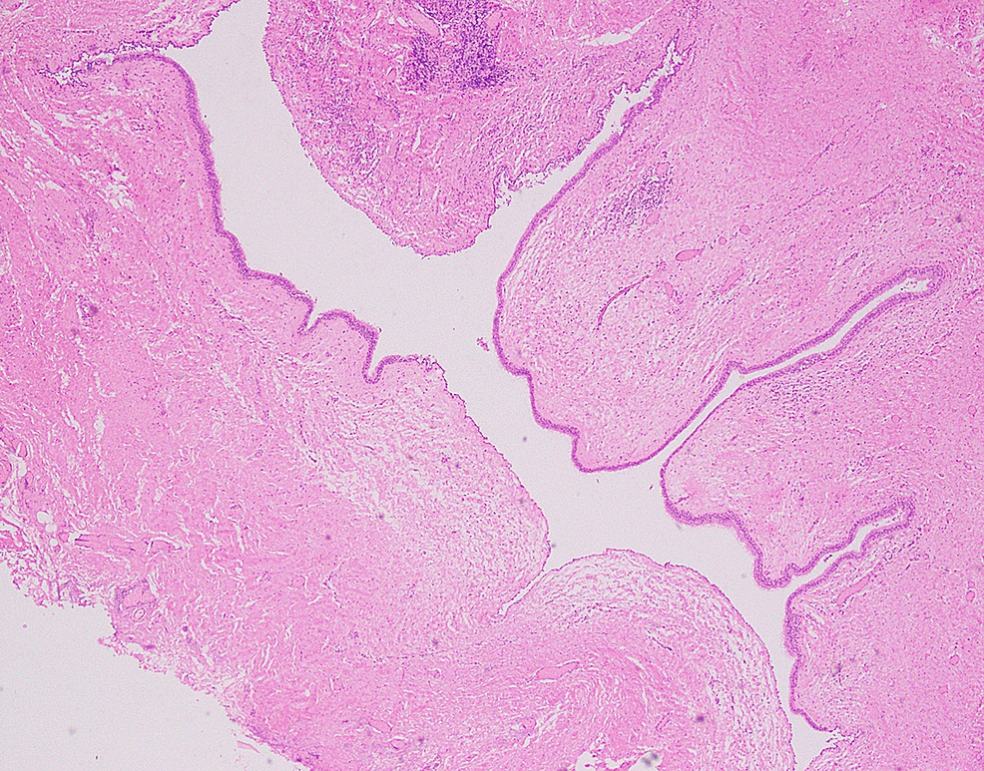
Low power view of cyst lined by ciliated epithelium with underlying fibrous cyst wall

Diagnosis of the bronchial cyst was an incidental finding on histopathology as it was not anticipated preoperatively and not recognized during surgery as it was mimicking as level VI lymph nodes. On histopathology, it was referred to as a bronchogenic cyst showing the lining thrown into small folds lined by ciliated lining and underlying fibrous cyst wall (Figures 2, 3).

## Discussion

Bronchogenic cysts are uncommon developmental anomalies of the tracheobronchial tree, and they rarely occur in the neck. More than 70 cases of bronchogenic cysts in the head and neck region have been reported in the literature so far [5]. Many bronchogenic cysts resemble metastatic lymph nodes and therefore required removal, dissection, and histopathological examination to be identified correctly [3].

Most frequently unilocular, they contain clear fluid or, less commonly, hemorrhagic secretions or air. These are lined by columnar ciliated epithelium, and their walls often contain cartilage and bronchial mucous glands [6]. It is unusual for bronchial cysts to have a patent connection with the airway, but when present, such communication may promote infection of the cyst by allowing bacterial entry [7].

The differential diagnosis for cervical bronchogenic cyst includes thyroglossal duct cyst, branchial cleft cyst, metastatic lymph node, thymic and thyroid cysts, dermoid cyst and lymphangiomas, cystic hygroma, teratoma, and cystic neuroma [8]. In our case, we could not differentiate the cyst from metastatic lymph node or other cystic lesions preoperatively and, of note, it was not differentiated intraoperatively as well.

Large cysts may cause dyspnea, respiratory distress, cough, and dysphagia in some individuals. Occasionally, secondary infection may occur, resulting in sinus tract formation, external drainage of purulent material if the cyst is superficial, or abscess formation if the cyst is deep [9].

A variety of cysts are seen in the head and neck region, and hence histopathological examination is essential for diagnosis. A typical bronchogenic cyst shows ciliated pseudostratified cuboidal to columnar epithelium with an underlying fibrous wall. Sometimes, variations may be seen, such as the presence of cartilage, seromucous glands, and smooth muscle [10]. However, in our case, none of these variations were found. The cyst showed a typical ciliated pseudostratified ciliated columnar lining. Hence, the diagnosis of the bronchogenic cyst was rendered.

Carcinomas arising from bronchogenic cysts have been reported in the literature [9]. These carcinomas arising from bronchogenic cysts emphasize the importance of total surgical excision [5].

## Conclusions

A bronchogenic cyst is a rare entity especially in the head and neck region, and it can often be confused with a metastatic lymph node. Diagnosis is made on the basis of histopathological examination, which requires surgical excision. The bronchogenic cyst should be considered in the differential diagnosis for midline and lateral neck masses.
